# A Systematic Literature Review of Information Sources for Threat Modeling in the Power Systems Domain

**DOI:** 10.1007/978-3-030-58295-1_4

**Published:** 2020-08-01

**Authors:** Engla Ling, Robert Lagerström, Mathias Ekstedt

**Affiliations:** 8 Department of Computer Science, University of Bristol, Bristol, UK; 9 Department of Computer Science, City, University of London, London, UK; Division of Network and Systems Engineering, KTH Royal Institute of Technology, Stockholm, Sweden

**Keywords:** Threat modeling, Power systems, Cyber security

## Abstract

Power systems are one of the critical infrastructures that has seen an increase in cyber security threats due to digitalization. The digitalization also affects the size and complexity of the infrastructure and therefore makes it more difficult to gain an overview in order to secure the entire power system from attackers. One method of how to gain an overview of possible vulnerabilities and security threats is to use threat modeling. In threat modeling, information regarding the vulnerabilities and possible attacks of power systems is required to create an accurate and useful model. There are several different sources for this information. In this paper we conduct a systematic literature review to find which information sources that have been used in power system threat modeling research. Six different information sources were found: expert knowledge, logs & alerts, previous research, system’s state, vulnerability scoring & databases, and vulnerability scanners.

## Introduction

Power systems are a crucial part of any country’s infrastructure. Without the access to electric power, the consequences can be severe, for example, hospitals being unable to operate. It is essential that the security of power systems is kept, especially when they are added to the communication infrastructure as part of digitalization. One method of ensuring the security of the power system is to gain an overview of its vulnerabilities by creating threat models. Threat modeling is a method of assessing security of systems and is further described in Sect. 3. Threat models are suitable for power systems because current security assessment techniques for enterprise IT environments might not be available for power systems. Power systems combine Information Technology (IT) with Operational Technology (OT) and there is often a combination of legacy and new techniques as well as equipment.

In this paper we find and discuss different information sources for vulnerability information when creating threat models for power systems. The aim is to contribute to the field of threat modeling in the power systems domain where finding reliable information to build a threat model is a crucial part of making sure that any conclusions drawn from the threat model is valid. It is not always clear how to find the information required to build a threat model and this article aims to aid in this process.

The research question is *What information sources have previously been used in research when generating threat models in the power systems domain?* The information sources have been found with a systematic literature review, i.e. a literature review where the process is carefully described so that the work can be repeated and avoid potential bias. This approach was chosen because there is a large amount of information in the research community, while in industry the created threat models are often not readily available being the potential security risk.

## Related Work

The related work most closely related to this paper is “A review of cyber security risk assessment methods for SCADA systems” [8]. In that paper “SCADA” and “risk assessment” were used as keywords to find articles between 2004 and 2014. The authors found 24 articles according to these criteria. The difference between that study and this one is that they focused on all types of SCADA systems and on risk assessment. Cyber security risk assessment can be considered a broader term than threat modeling because the risk can be assessed by other means than with threat modeling. For example, it can be assessed by mathematical formulas. Another difference between cyber security risk assessment and threat modeling is that in risk assessment, the consequences of an attack are also taken into consideration. Even though the focus of that paper was not information sources, they presented it as part of their results. The authors found two different possible information sources, expert opinion and historical data. This is in contrast to this literature review paper where historical data was not mentioned in any of the articles and many more information sources were found. In addition, the different potential information sources were discussed in the article, indicating that the authors were aware that more than the two found by them exists.

Nazir et al. performed a survey for the techniques used to assess vulnerabilities of SCADA system [32]. Their survey was not a systematic literature review, but rather a collection of the most commonly used techniques including threat modeling. Some of the techniques that they discuss can be considered as information sources, for example using monitoring and test beds. They also discuss tools that can be used for gathering information. These tools are scanning tools, penetration testing, machine learning, Intrusion Detection System (IDS), Intrusion Prevention System (IPS), honey pots, Security information and event management (SIEM), ethical hacking and forensic science.

## Threat Modeling

Threat modeling has many different definitions [45], but it is typically defined as a technique where the different security threats for a system are modelled. Often they are modelled in order to run simulations. This allows you to find potential future attacks. In this way one can make sure that the system is secure by using a model of the system instead of the actual system.

There are multiple ways of how to represent a threat model. For example, attack trees that were made popular by Schneier [37] shows a successful attack as the goal node of a tree with different attack steps leading up to that goal node. According to his paper, the attack goals, attacks against those goals and the values of the nodes, that is, the difficulty of the attacks, are required to build the attack tree. This type of information to build attack trees or any other type of threat model can be found by using several different information sources. For example, one can derive the information from vulnerability databases, industry experts or base it on previous attacks. It is important that this information source is reliable so that the resulting threat model can be trusted. The threat model may be used to make decisions of where to assign the most resources to protect the system depending on the result and any mistakes could be costly.

## Method

The scope of this systematic literature review is defined by the search query that was used and the databases that were searched. The keywords were chosen based on the best of our knowledge on what would be the most inclusive but also include relevant articles. The following search query with keywords was used: (“attack graph” OR “attack tree” OR “threat model*”) AND (“power system” OR “energy system” OR “power grid” OR “smart grid”). The asterisk symbol means that the query will search for multiple variations of threat model, including “threat models” and “threat modeling”.

The databases IEEE, Springer link, Web of Science, Science Direct and Scopus were used in this systematic literature review. In total 260 articles were found, and the specific search queries with results are shown below. The search was performed on the January 24th 2020.

**IEEE** 76 results after a search by Metadata. The metadata consists of abstract, title and indexing terms. Indexing terms are keywords that have been defined by the author.

**Springer Link** 415 results that were manually narrowed down to 17 results. This was because the title, abstract and keywords had to be filtered manually since Springer Link does not support that search request.

**Web of Science** 45 results after searching based on Topic, which includes title, abstract, author keywords, and Keywords Plus. Keywords plus are keywords generated automatically by an algorithm. The algorithm creates keywords based on the titles of the articles references.

**Science Direct** 9 results found based on Title, abstract or author-specified keywords.

**Scopus** 113 results based on their “TITLE-ABS-KEY” search.

After duplicates were removed from the final 260 articles, 146 of them remained. These articles were narrowed down to 44 articles according to the following inclusion criteria:

- The article must focus on cyber security.
- The article must include a threat model or the method of how one was constructed.
- The threat model must be in the power systems domain.
- The article must mention at least one information source that was used to create the threat model.
- The article must be written in English.

When the final 44 articles had been found, they were divided into categories of information source.

## Information Sources

In Table 1 the information sources that were found in each article of the systematic literature review is summarized. This section describes and discusses the different information sources as found by this systematic literature review.

### Expert Knowledge

From the results, 11 threat models were generated by using expert knowledge. Expert knowledge means that the developers have gained information from experts regarding, for example, what they believe to be the most likely attack scenarios. These experts are not necessarily experts in security but they are rather experts in the power systems domain. There is one exception and that is one article that used hacking knowledge [15]. The information was found by reading about attackers describing their goals.

Most often there is little information regarding the experts, how many they are and their credentials [25, 36]. This makes it difficult to validate their results. Even if the experts are well known in terms of their credentials it can be difficult to know how much to stress each expert’s opinion. In an article outside the scope of this review [22] the authors analyzed how to evaluate the correctness in expert knowledge judgement and how this could be used to weight in how much one considered a specific expert’s opinion. This opens up the possibility of using expert knowledge, while prioritizing knowledge put forward by reliable experts.

There are some articles that discuss the method itself and these methods are the Delphi method [6, 7], brainstorming [40] or allowing the experts to model themselves [18]. The Delphi method is a process of anonymous scoring followed by open discussions and results iteratively. The authors that used the brainstorming method saw a possible weak spot because of its dependency on who is participating from the stakeholders [40]. However, they claim that this was partly helped by the attending security experts. In the paper where the experts were allowed to model themselves they had some issues because the expert’s modeling was not always in line with what could be translated easily to the threat modeling framework that they used [18].

One of the limitations of using industry experts is that they are often experts within one specific domain. This might make it difficult to model larger systems. This limitation was mentioned by [41]. In addition, they mentioned two other limitations. These are that the results are only valid during that instance and are constrained by the experts knowledge level. The authors expert knowledge reference group consisted of utility sector experts of Operational Technology (OT) systems. The experts were used to model the data flows. However, other information sources were used to create their attack graph because attack data was included in the framework that they used. In the threat modeling technique by Chen et al., a suggestion to this problem is presented where multiple smaller threat models are combined into one larger [5]. Each of the smaller threat models would be created by an industry expert in that domain.

Some articles use expert knowledge in combination with other information sources [15, 17, 41]. It can be easy to include expert knowledge because it may only be a question of asking an expert of their opinion. As seen in the articles of this systematic literature review, there are many different methods of how to include expert knowledge. This supports the fact that expert knowledge is easy to modify and adapt to your needs. However, one can question how to validate the results.

### Logs and Alerts

A few articles use logged information as an information source. There are many different services that collect information about a system as logs and the resulting articles includes three of these different kinds of logs. One paper used information from archived IDS/IPS logs and alerts [15]. Another paper used event logs that they created themselves by running simulated attacks [14]. This might require a lot of time and skill to set up. An alternative could be to use historical data logs if that is available as Zhang et al. did [50]. Logs can be a very good source of information because there is often a very large amount of data that is collected. At the same time, it can be time consuming and require knowledge to go through all of the information.

**Table 1.**

Information sources for power system’s threat models

### Previous Research

The majority of articles found in this systematic literature review use previous research to find the different possible vulnerabilities or attacks on the systems that they model [4, 13, 16, 19, 23, 26, 28–30, 34, 46, 52, 53]. Previous research is also used in combination with other information sources [9–11, 20, 31, 41]. This previous research can consist of research articles or published reports. In most cases it is not exactly specified where the authors have used previous research and where they have made assumptions. This makes it difficult to validate their results. Previous research is however a valid information source where it is referenced properly because the source has often been peer-reviewed before publication.

### System’s State

Five of the articles used the state of the system that they want to model to evaluate the vulnerabilities or potential attacks. The state of the system can be to for example analyze the security implementations [15, 27, 38, 39], make a forensics analysis [38, 39] or look at configuration files [20].

There was a large variation of the type of information used from the system and how they used it found in this review. When using a system’s state as an information source, no other resource or information is required except the system itself. This makes the information source easy to use. However, it might take time to collect the appropriate information and experts to be able to analyze the results accurately.

### Vulnerability Scoring and Databases

17 articles of this systematic literature review was found to use vulnerability scoring or databases as an information source. Some of the articles use a combination of the scoring systems with the databases and some only use one of them. The most common scoring system as found in this review is the Common Vulnerability Scoring System (CVSS)^1^ [42, 43, 47, 48, 51]. The CVSS is a scoring system that was developed by and maintained by Forum of Incident Response and Security Teams (FIRST). The authors in [21], outside the scope of this review, found that using CVSS alone does not perform well as an information source for threat modeling. While the authors in [24] found that in general CVSS can be trusted. In addition, their results also showed that if one considers all of the systems vulnerabilities and not only those with the highest CVSS score, the results were much more reliable. Some papers found in this review use a combination of calculating their own CVSS scores with vulnerability databases or other information sources [9–11]. Another example of a scoring system is the Common Weakness Enumeration (CWE) is a category system that also calculates a score for the weaknesses or vulnerabilities^2^ [35]. The CWE includes all software weaknesses and not only vulnerabilities.

There are multiple different vulnerabilities databases that aim to categorize and explain vulnerabilities for different systems and these can be used as information sources when modeling. The ones found in this review are the U.S. National Vulnerability Database (NVD)^3^ [2, 12, 17, 41, 49], MITRE^4^ [31, 44], several CERTs databases^5^^,^^6^^,^^7^ [1, 12, 44] as well as the Chinese databases Chinese National Vulnerability Database (CNNVD)^8^ [41].

The positive aspect of using this information as a source is that a lot of information is readily available. This information source can also adapt easily to changes. If one is constructing a threat model with this source, it is possible to quickly update the vulnerability information if the scoring or database change. One downside of using the vulnerability scoring and databases is that this information has the possibility of bias. This is because there are experts who use their experience and knowledge to estimate properties that give vulnerabilities a specific score. It is difficult to know exactly where the articles gain the information, they might phrase it as “vulnerabilities can be found in vulnerability databases, for example X,Y,Z”. In these cases we assume that those are the databases used.

### Vulnerability Scanner

A vulnerability scanner can automatically detect vulnerabilities within a system and three articles in this literature review used them as an information source [2, 17, 35]. In all of these papers, the vulnerability scanner was used in combination with another information source. A scanner may require time to set up, but it provides a lot of information that can often already be analyzed by the tool itself. Similar to the information source of previous research, it is not always clear to what extent the vulnerability scanning has been used. Sometimes the articles will give examples of vulnerability scanning tools that may be used, and in this can the assumption is that those tools have actually been used.

## Discussion

Similar to any systematic literature review work, there is the possibility of changing the database, keywords and inclusion criteria that is used. For example, Google Scholar and arXiv could have been searched as well. The downside of using a database, such as Google Scholar, is that it includes other work such as e.g. student thesis reports in addition to peer-reviewed research papers. If we would search the full text of articles and not only the title, abstract and keywords then the result does not only show many more articles, but these articles may not be relevant for the research question.

Another limitation of this work that exists because of the nature of a systematic literature review is that some articles will not be included in the results. For instance, the terms “risk assessment” [33], “SCADA” [3] may be used instead of the search terms in this systematic literature review. However, if those search terms were included the review would result in many irrelevant articles. There may also be other information sources being used other than those included in published research. These might be information sources used by threat models created in the industry.

Possible future work includes looking into the relations between the different information sources and how some of them may overlap or complement each other. It could also be to look into information sources that exist in other domains and use these in the power systems domain. Future work could also be to automatically keep this list of information sources up to date by utilizing the databases’ API and some form of natural language processing solution. Planned future work is to use some of the found information sources to develop threat models for power systems.

## Conclusion

In this article a systematic literature review was used as a method to find the different information sources that have previously been used in research when creating threat models in the power systems domain. Answering the research question, this systematic literature review found six different information sources. These are expert knowledge, logs & alerts, previous research, system’s state, vulnerability scoring & databases and vulnerability scanners.

The many different information sources that have been used indicate that there is no standard method that is usually followed. The most common information sources that are used are expert knowledge, previous research, and vulnerability scoring & databases. As discussed in this paper there are many positive and negative aspects with all of the information sources. Because of the availability of information for these sources it might be beneficial to combine all three when creating a threat model in the power systems domain.

## Footnotes

1 https://www.first.org/cvss/ [accessed 28 April 2020].

2 https://cwe.mitre.org/ [accessed 28 April 2020].

3 https://nvd.nist.gov/ [accessed 28 April 2020].

4 https://cve.mitre.org/cve/search_cve_list.html [accessed 28 April 2020].

5 https://www.kb.cert.org/vuls/search/ [accessed 28 April 2020].

6 https://www.cert.org.cn/ [accessed 28 April 2020].

7 https://www.us-cert.gov/ics/monitors [accessed 28 April 2020].

8 http://www.cnnvd.org.cn/ [accessed 28 April 2020].
